# Morphological Transformation between Nanocoils and Nanoribbons via Defragmentation Structural Rearrangement or Fragmentation-recombination Mechanism

**DOI:** 10.1038/srep27335

**Published:** 2016-06-02

**Authors:** Yibin Zhang, Yingxuan Zheng, Wei Xiong, Cheng Peng, Yifan Zhang, Ran Duan, Yanke Che, Jincai Zhao

**Affiliations:** 1 Beijing National Laboratory for Molecular Sciences, Key Laboratory of Photochemistry, Institute of Chemistry, Chinese Academy of Sciences, Beijing 100190, China; 2 University of Chinese Academy of Sciences, Beijing 100049, China

## Abstract

Kinetic control over the assembly pathways towards novel metastable functional materials or far-from-equilibrium systems has been much less studied compared to the thermodynamic equilibrium self-assembly. Herein, we report the distinct morphological transformation between nanocoils and nanoribbons in the self-assembly of unsymmetric perylene diimide (PDI) molecules. We demonstrate that the morphological transformation of the kinetically trapped assemblies into the thermodynamically stable forms proceeds via two distinct mechanisms, i.e., a direct structural rearrangement (molecule 1 or 2) and a fragmentation-recombination mechanism (molecule 4), respectively. The subtle interplay of the steric hindrance of the bulky substituents and the flexibility of the linker structure between the bulky moiety and the perylene core was demonstrated to enable the effective modulation of the energetic landscape of the assemblies and thus modulation of the assembly pathways. Herein, our work presents a new approach to control the self-assembly pathways and thereby can be used to achieve novel far-from-equilibrium systems.

Equilibrium self-assembly via the interplay of various interactions, including hydrogen bonding, π-π interactions, hydrophobic interactions, electrostatic interactions, steric effects, and among others, has been well understood and documented^1^,^2^,^3^,^4^,^5^,^6^,^7^,^8^. In contrast, kinetic self-assembly towards far-from-equilibrium systems, particularly kinetic control over growth pathways and understanding of the mechanism have been much less studied^9^,^10^,^11^,^12^,^13^,^14^,^15^,^16^. In recent years, the research area of kinetic self-assembly has advanced remarkably and some impressive examples, including the generation of metastable assemblies with extraordinary properties and the creation of living supramolecular polymerization via control over pathway complex, have been reported^14^,^15^,^17^,^18^,^19^. In these systems, the transformation of the metastable assemblies into the thermodynamically stable forms driven by the energy-landscape occurred spontaneously via either a fragmentation-recombination mechanism or a simple structural rearrangement (defragmentation)^9^,^15^,^17^. The fragmentation-recombination process plays a critical role in the generation of the living supramolecular self-assembly^17^, while the direct structural rearrangement gives a significant insight into the nature of molecular self-assembly^10^. However, regarding the molecular origin of the specific mechanism in the resulting morphological transition, the available information has been very limited. In this context, we studied the self-assembly pathways of four unsymmetric perylene diimide (PDI) molecules (Fig. 1) that exhibited spontaneous morphological transformation between nanocoils and nanoribbons. Our molecular design is based on the bulky substituents on different positions of phenyl moiety in one side chain of PDI molecules (that tunes the steric hindrance) and the different linkers (that provides the flexibility for molecular rearrangement) between the same phenyl moiety and the perylene core. By employing electron microscopy, confocal optical microscopy, and time-dependent optical characterizations, we demonstrated that morphological transformation of **1** and **2** assemblies proceeded quickly through the direct structural rearrangement (defragmentation) due to the relatively small steric hindrance resulting from the 3-methoxybenzyl ether substituent on the meta- position of phenyl moiety in molecules **1** and **2** (Fig. 1). By contrast, the initially formed nanocoils from **3** underwent no morphological changes (Fig. 1) due to the increased steric hindrance from the 3-methoxybenzyl ether substituent on the ortho-position of phenyl moiety and relatively low flexibility of the methylene linker. Interestingly, when using the ethylene linker instead of the methylene linker to increase the molecular flexibility, spontaneous morphological transformation of **4** nanocoils into elongated nanoribbons occurred through a fragmentation-recombination mechanism (Fig. 1). Here, the application of the interplay of the steric hindrance of the bulky substituents and the flexibility of a linker between the substituted phenyl moiety and the perylene core opens a new path to control the self-assembly pathways and thereby has the potential to achieve novel systems far from thermodynamic equilibrium.

The detailed synthesis, characterization, and self-assembly of molecules **1–4** are described in the Supporting Information. Design of these unsymmetric PDI molecules is based on the substituted position of the 3-methoxybenzyl ether group on the phenyl moiety in one side chain of PDI molecules and the linker structure between the same substituted phenyl moiety and the perylene core. The different substituted position of the 3-methoxybenzyl ether group on the phenyl moiety enable the generation of the distinct steric hindrance for the intermolecular rearrangement, while the linker structures can modulate the flexibility of molecular rearrangement^20^. In a typical self-assembly process, on flash injection of the 0.3 mL chloroform solution of the corresponding molecules (0.65 mM) into 4.5 mL ethanol, the resulting mixture was immediately stirred and then allowed to stand at room temperature for certain duration. The time-dependent absorption and fluorescence spectral changes were used to monitor the self-assembly process. As shown in Fig. 2a, a new absorption band centered at 573 nm immediately emerged after the self-assembly of **1**, which was gradually increased until 4 h and remained unaltered afterwards. Because the amount of the monomers left after 5 min self-assembly is only ca. 0.3% of the initial amount of the monomers (determined through comparison of the fluorescence intensity before and after the self-assembly), the assembly of the monomers into the aggregates after 5 min is negligible and the subsequent absorption changes at 573 nm suggest the transformation of an aggregation to another. The morphological transformation of an aggregation into another was clearly reflected by the changes in the fluorescence spectra. Obviously, there is no fluorescence overlap between the monomer and the aggregates and the blue-shifted fluorescence bands from 662 nm to 652 nm reflect the morphological transformation from an aggregation into another. Notably, the fluorescence intensity also decreased with time, which is consistent with the fluorescence quantum yields of the two aggregations (36% and 29%, respectively), indicative of the molecular reorganization during the morphological transformation. Transmission electron microscopy (TEM) images confirmed the initial formation of nanoribbons accompanied by a small number of nanocoils after 5 min self-assembly of **1** (Fig. 2c). As time progressed (e.g., 1 h later), more nanocoils were formed, which is accompanied with the decreasing of the nanoribbons (Fig. 2c). After 6 h, the nanoribbons completely disappeared and only the nanocoils were observed (Figs 2c and S1). The nanocoils with the pitch length of ca. 50 nm remained unchanged even after weeks of suspending in solution, indicating the thermodynamic nature of the nanocoils. The high fluorescence quantum yield (ca. 36%) of initially formed nanoribbons suggests that molecule **1** adopts a slipping stacking with a relatively large transversal offset within the nanoribbons^21^. Such molecular arrangement has been demonstrated to be simultaneously favorable for π-overlap and emission^22^,^23^. During the morphological transformation from the nanoribbon to the nanocoil, molecule **1** in a slipping stacking rotates into a helical stacking, which reduces the π-overlap between **1** molecules and thereby results in the blue-shifted fluorescence in the resulting nanocoils (Fig. 2b). Notably, in the process of the morphological transition of nanoribbons to nanocoils, negligible changes of their length were observed. This observation is further confirmed by the confocal laser scanning optical microscope (CSOM) which was used to monitor the self-assembly process. Despite the relatively low resolution of the imaging (Fig. 2d), the length scale of the nanoribbons and nanocoils at different assembling time points is shown to be similar to each other. Closer examination of the assemblies in the intermediate time points (e.g., after 1 h) by TEM revealed the formation of the intermediate morphology with partly coiled structure (Fig. S2), suggesting that a merely winding structural rearrangement resulted in the transformation of the kinetic nanoribbons into the thermodynamically stable nanocoils. To further confirm the direct transformation of the nanoribbons into nanocoils rather than through a fragmentation-recombination mechanism as observed in the formation of the amyloid fibril^24^,^25^, we investigated the effect of the initial concentration of **1** on the dynamic transformation behavior. As shown in Figs 2a and S3, the transformation rate of morphological changes was not dependent on the initial concentration of **1** and all morphological changes were finished in 4 h, confirming the directly winding transformation of nanoribbons into nanocoils^17^. This result is in sharp contrast to the initial concentration-dependent fragmentation-recombination mechanism^14^,^17^,^26^.

Interestingly, molecule **2**, bearing the ethylene linker, initially formed the nanocoils (Fig. 3c) rather than the nanoribbons upon flash injection of the 0.3 mL chloroform solution of **2** (0.65 mM) into 4.5 mL ethanol and immediate stir. After 2 h, most nanocoils transformed into the nanoribbons and the total transform process finished after 4 h when only nanoribbons with the width of ca. 25 nm and length of several micrometers were observed (Fig. 3c). Likewise, the length scale of the nanocoils and nanoribbons formed at different assembling time points remains alike, as shown in Fig. 3c,d. The intermediate morphology with partly ribbon structure was also observed (Fig. S4), indicating that a merely unwinding rearrangement resulted in the transformation of the kinetic nanocoils into the thermodynamically stable nanoribbons, which well explained the alike length scale above mentioned. The merely unwinding of nanocoils into nanoribbons was further supported by the facts where the transformation rate of morphological changes (the absorption changes at 590 nm were finished in 4 h) was not dependent on the initial concentration of **2** (Figs 3a and S5). Such unwinding transformation was also reflected by the time-dependent fluorescence spectra profiles. As shown in Fig. 3b, an emerged emission band centered at 654 nm during the initial stage of self-assembly was slightly red-shifted with time until 4 h. This result, together with the fact that the fluorescence intensity decreased (the fluorescence quantum yield decreased from 16% to 11%), reflects that a helical packing of **2** within the nanocoil gradually transforms into a slipping packing with a small transversal offset (a longitudinal slipping usually results in the pretty low emission efficiency and thus is ruled out) within the resulting nanoribbon. Here, what is noteworthy is that such a small chemical modification of the linker structure of **2** reverse the energy landscape of the off-pathway (nanocoils) and on-pathway (nanoribbons) compared to the case of **1** where the off-pathway and on-pathway assemblies were nanoribbons and nanocoils, respectively.

The influence of the linker structure on the energetic landscape of the on-pathway and off-pathway assemblies as observed above motivated us to investigate the effect of the increased steric hindrance on the self-assembly pathway. To this end, molecule **3** that has an increased steric hindrance because of the 3-methoxybenzyl ether substituent on the ortho-position of the phenyl moiety compared to **1** and **2** was synthesized. Under the identical assembly conditions used above, well-defined nanocoils with pitch length ca. 50 nm were observed to be formed, as shown in Fig. 4c. Notably, the resulting nanocoils from **3** did not transform into other structures (Fig. 4c), which is distinct from the case of molecules **1** and **2**. We postulate that the increasing steric hindrance in **3** disfavored the dynamic molecular rearrangement required for the morphological transformation. The avoidance of molecular rearrangement in the **3** nanocoils was supported by the time-dependent optical spectra profiles where the optical spectra remain almost the same in the self-assembly process (Fig. 4a,b). Further evidence comes from the fact that the fluorescence quantum yield of the resulting nanocoils (46%) remain unchanged during the assembly process. The freeze of the morphological transformation by the strong steric hindrance in this work is reminiscent of the porphyrin-based system^27^.

Interestingly, when the methylene linker in **3** was replaced with the ethylene linker to afford molecule **4,** the enhanced flexibility of the molecule re-enabled the morphological transformation during the identical self-assembly process. Like molecule **2**, molecule **4** formed the nanocoils at the initial self-assembly, which were slowly transformed into the nanoribbons (Fig. 5a). However, unlike the case of molecule **2** where the length scale of the nanocoils and nanoribbons remained the same, the length of **4** nanoribbons formed after the transformation was greatly increased compared to that of the initially formed nanocoils (Fig. S6). The grown nanoribbons with different length at different assembly time points were further imaged by the CSOM (Fig. 5b). These results suggest that the formation of **4** nanoribbons was proceeded via a nucleation-elongation mechanism rather than a mere structural rearrangement from the nanocoil precursor as observed in **1** and **2** assemblies. To corroborate the mechanism of the morphological transformation of **4**, we carefully examined the assemblies at the intermediate time points (e.g., at 1 h) by TEM. As shown in Fig. S7, shorter nanocoils appeared after 1 h self-assembly, accompanied with the appearance and growth of the nanoribbons, indicative of the fragmentation of the nanocoils to the stock monomers for the elongation of the nanoribbons (Fig. 1). Because of the distinct molecular organization within the nanocoil and nanoribbon, the fluorescence quantum yields of the nanocoil and nanoribbons are 39% and 32%, respectively. Furthermore, the time-dependent absorption spectra profile confirmed that the morphological transformation of **4** nanocoils into nanoribbons was greatly dependent on the initial concentration of **4** (Fig. S8); the time required for the complete transformation into nanoribbons became much longer as the initial concentration of **4** increased (Figs 5c–e and S8). This inverted dependence indicates that the nanoribbons were elongated by recruiting the **4** monomer resulting from the fragmentation of the metastable nanocoils (Fig. 1). These results above allow us to conclude that the subtle interplay of the steric hindrance and molecular flexibility can effectively modulate the assembly pathways (Fig. 1), which has the potentials in establishing systems that are far from thermodynamics equilibrium.

In conclusion, we report the distinct spontaneous morphological transformation between nanocoils and nanoribbons in the self-assembly of unsymmetric PDI molecules. Two mechanism for the distinct assembly pathways, i.e., a mere structural rearrangement as exemplified by molecule **1** or **2** and a fragmentation-recombination mechanism as exemplified by molecule **4**, were determined. The subtle interplay of the steric hindrance of the substituents on the phenyl moiety in one side chain and the flexibility of the linker structure between the same phenyl moiety and the perylene core was demonstrated to enable the effective modulation of the energetic landscape of the assemblies and thus modulation of the assembly pathways. Our work herein provides a new approach to control the self-assembly pathways and thereby has the potential to achieve novel systems far from thermodynamic equilibrium.

## Methods

### Self-assembly of molecules 1–4

Molecules **1**, **2**, **3,** and **4** were self-assembled by injecting a chloroform solution (0.3 mL) of the corresponding compound (0.06 mM, 0.13 mM, 0.26 mM, or 0.65 mM) into 4.5 mL of ethanol (poor solvent) in a test tube or vial followed by full mixing and aging. The assemblies suspended in solution at different assembly time points can be easily transfered onto various substrates by simple drop-casting or spin-coating for characterizations. The self-assembly process was monitored and recorded by the combination of optical spectroscopy and electronic microscopy.

### Structural and property characterization

UV-vis absorption spectra were measured on a PerkinElmer Lambda 35. Fluorescence spectra were obtained on a PerkinElmer LS 55 luminescence spectrophotometer with an excitation wavelength of 450 nm. All the optical spectra were measured over suspensions in the mixture of ethanol and chloroform (volume ratio, 15:1). The fluorescence quantum yields of the aggregates were determined by the integrating sphere method performed using a Hamamatsu Absolute PL Quantum Yield spectrometer C11247. Various excitation wavelengths ranging from 450 to 570 nm were employed for the fluorescence quantum measurements of each aggregate, and the variance of the fluorescence quantum yield is at the range of ±2%. Transmission electron microscopy (TEM) measurements were performed with a JEM-2011 (120 KV). Optical and fluorescent images were recorded by confocal laser scanning microscopy using objective lens (Olympus, UPLSAPO 100 XO; 1.40 NA, 100×). The pictures were recorded in fluorescence tunnel using 488 nm laser (3.6 W/mm^2^, 4 us/pixel, 800 × 800 pixels/frame).

## Additional Information

**How to cite this article**: Zhang, Y. *et al.* Morphological Transformation between Nanocoils and Nanoribbons via Defragmentation Structural Rearrangement or Fragmentation-recombination Mechanism. *Sci. Rep.* **6**, 27335; doi: 10.1038/srep27335 (2016).

## Supplementary Material

Supporting Information

## Figures and Tables

**Figure 1:**
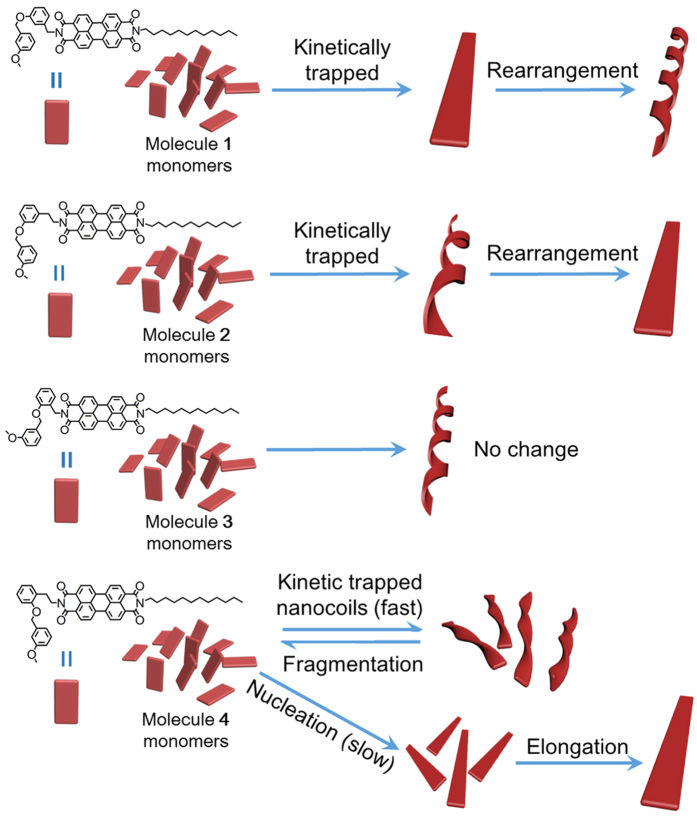
Schematic representation of the morphological transformation of assemblies from molecules 1–4.

**Figure 2:**
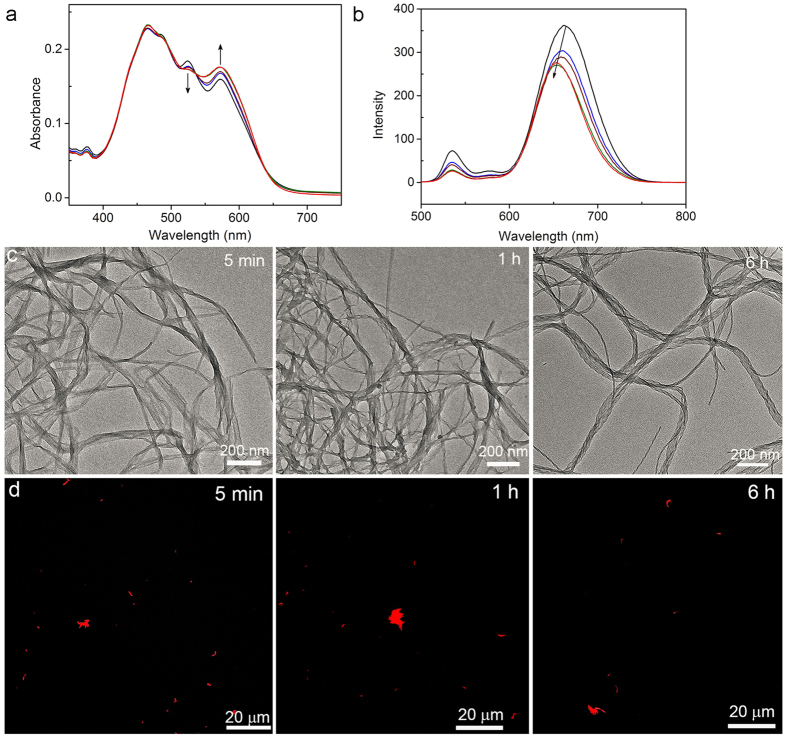
(**a**) Absorption and (**b**) fluorescence spectra profile (excited at 450 nm) of the assembles from **1** at different assembling time points: 5 min (black), 30 min (blue), 1 h (wine), 4 h (olive), and 15 h (red). (**c**) TEM imaging of the assemblies from **1** at different self-assembling time points. (**d**) CSOM imaging of the assemblies from **1** at different self-assembling time points.

**Figure 3:**
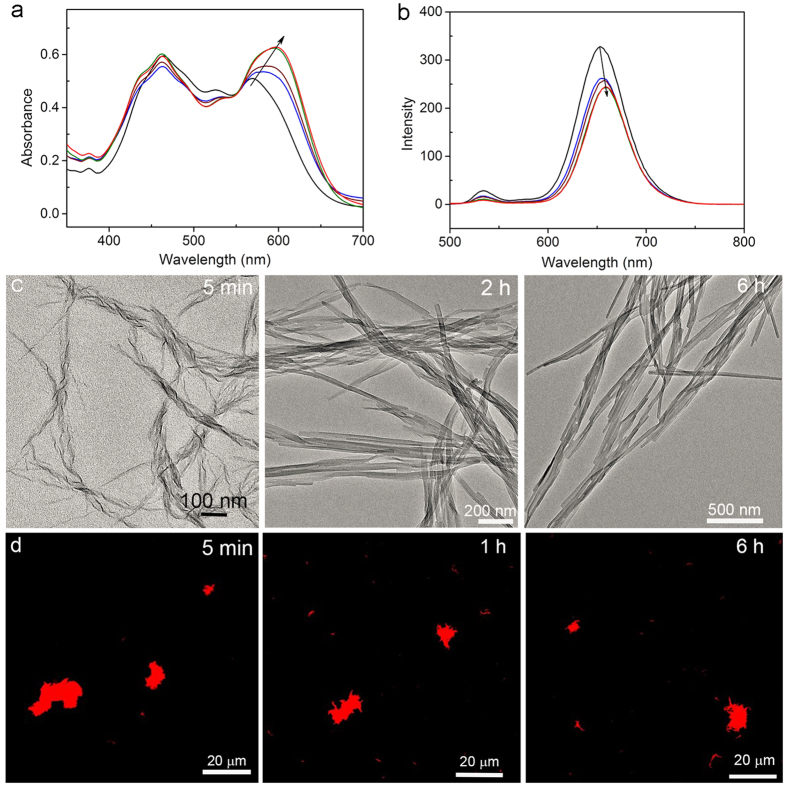
(**a**) Absorption and (**b**) fluorescence spectra profile (excited at 450 nm) of the aggregates from **2** at different assembling times: 5 min (black), 30 min (blue), 1 h (wine), 4 h (olive), and 19 h (red). (**c**) TEM imaging of the assemblies from **2** at different self-assembling time points. (**d**) CSOM imaging of the assemblies from **2** at different self-assembling time points.

**Figure 4:**
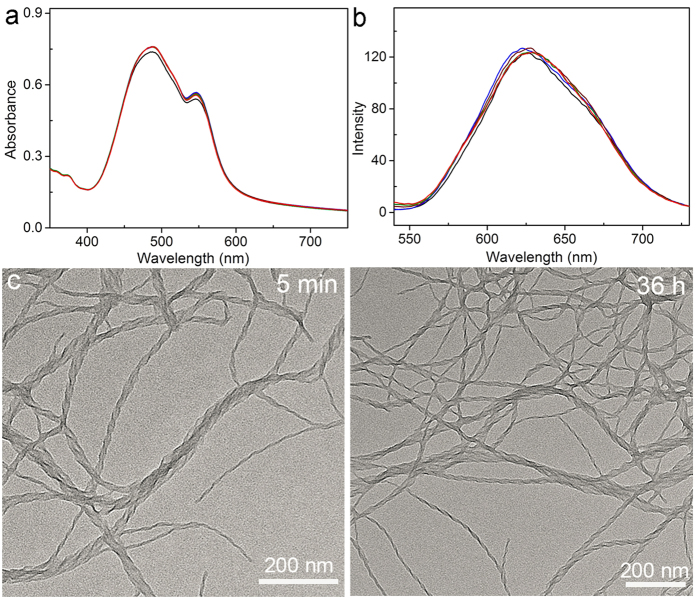
(**a**) Absorption and (**b**) fluorescence spectra profile (excited at 450 nm) of the aggregates from **3** at different assembling times: 5 min (black), 30 min (blue), 1 h (wine), 4 h (olive), and 24 h (red). (**c**) TEM imaging of the assemblies from **3** at different self-assembling time points.

**Figure 5:**
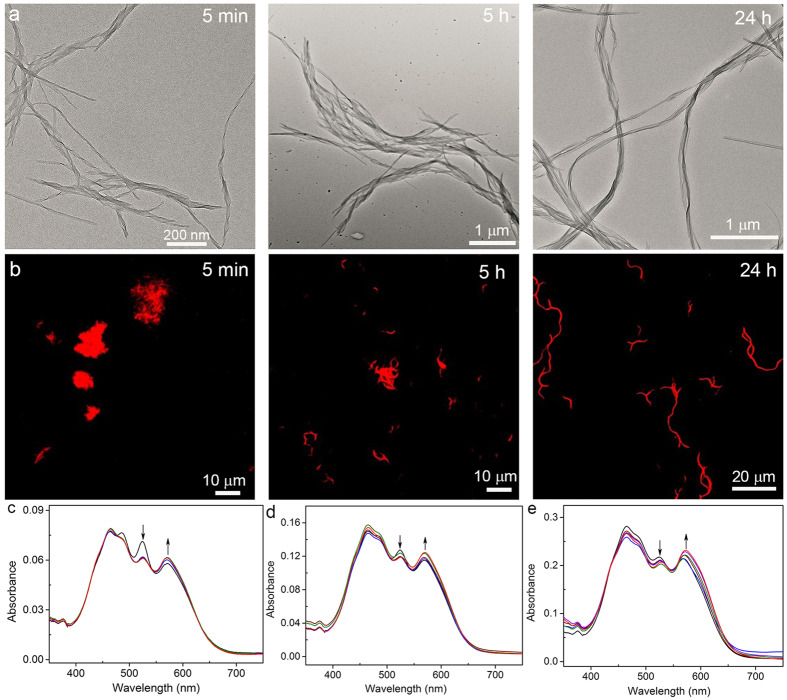
(**a**) TEM imaging of the assemblies from **4** at different self-assembling time points. (**b**) CSOM imaging of the assemblies from **4** at different self-assembling time points. (**c**) Absorption spectra profile of **4** aggregates at different assembly times after the injection of 0.3 mL of chloroform solution of **4** with 0.06 mM into 4.5 mL of ethanol: 5 min (black), 30 min (blue), 1 h (wine), 3 h (olive), and 24 h (red). No further absorption change after 1 h (see Fig. S8). (**d**) Absorption spectra profile of **4** aggregates at different assembly times after the injection of 0.3 mL of chloroform solution of **4** with 0.13 mM into 4.5 mL of ethanol: 5 min (black), 1 h (blue), 5 h (wine), 8 h (olive), and 24 h (red). No further absorption change after 8 h (see Fig. S8). (**e**) Absorption spectra profile of **4** aggregates at different assembly times after the injection of 0.3 mL of chloroform solution of **4** with 0.26 mM into 4.5 mL of ethanol: 5 min (black), 1 h (blue), 8 h (wine), 24 h (olive), 34 h (violet), and 48 h (red). No further absorption change after 34 h (see Fig. S8).
