# Supplementary material for: Morphological Transformation between Nanocoils and Nanoribbons via Defragmentation Structural Rearrangement or Fragmentation-recombination Mechanism
Source: Sci Rep. 2016 Jun 2;6:27335. doi: 10.1038/srep27335 (PMC4890316; doi:10.1038/srep27335)
Supplement: Supporting Information [file srep27335-s1.pdf]

Supporting Information

Morphological Transformation between Nanocoils and Nanoribbons via Defragmentation Structural Rearrangement or Fragmentation-recombination Mechanism

Yibin Zhang‡, Yingxuan Zheng‡, Wei Xiong, Cheng Peng, Yifan Zhang, Ran Duan, Yanke Che*, Jincai Zhao

Beijing National Laboratory for Molecular Sciences, Key Laboratory of Photochemistry, Institute of Chemistry, Chinese Academy of Sciences, Beijing 100190, China.

Email: ykche@iccas.ac.cn

Materials

All reagents and solvents were obtained from commercial suppliers and used as received unless otherwise noted.

Other supplies: Fisherfinest Premium Cover Glass (25×25×0.17 mm) purchased from Fisher Scientific. Filter membrane (Teflon 0.45 μm) purchased from PerkinElmer.

General procedure for synthesis of molecules 1-4.

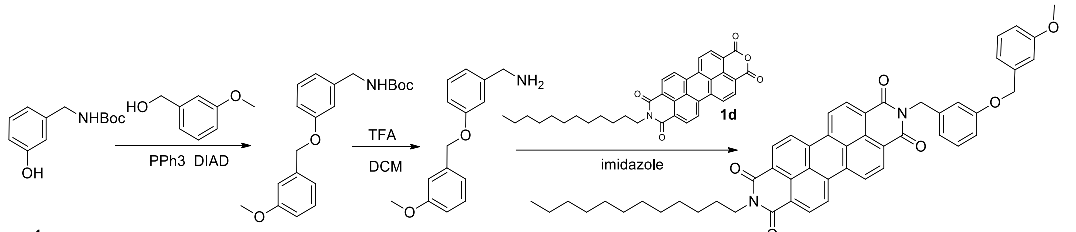

Tert-butyl 3-((3-methoxybenzyl)oxy)benzylcarbamate (1b). 1a[1] (500 mg, 2.24 mmol), 3-Methoxybenzyl alcohol (309 mg, 2.24 mmol), Diisopropyl azodicarboxylate (DIAD) (542 mg, 2.69 mmol), PPh_3 (704 mg, 2.69 mmol) were added into 10 mL dichloromethane and stirrerfd overnight at room temperature under N_2. Then the reaction was added with H_2O (10 mL) and the mixture was extracted with dichloromethane (3 × 10 mL). The combined organic layers were dried over sodium sulfate, and concentrated under vacuum. The residue was purified by column chromatography (silica, hexanes: ethyl acetate = 10:1) to afford 1b (470 mg).

^{1}HNMR (400 MHz, $CDCl_3$): δ 7.43-7.12 (m, 2H), 7.01-6.98 (m, 2H), 6.88-6.82 (m, 4H), 5.02 (s, 2H), 4.84 (br, 1H) 4.28 (s, 2H), 3.80 (s, 3H), 1.46 (s, 9H).

(3-((3-methoxybenzyl)oxy)phenyl)methanamine (1c). 1b (470 mg) was added to a solution of dichloromethane (5 mL) and trifluoroacetic acid (5 mL). The resulting solution was stirred for 3 h at room temperature, and then trifluoroacetic acid was removed by rotary evaporation. Saturated $NaHCO_3$ solution (10 mL) was added to the residue and the resulting solution was extracted with dichloromethane (3 ×10 mL). The combined organic layers were dried over sodium sulfate, and concentrated under vacuum to afford compound 1c**,** which was not purified and used directly for the

following reaction.

Molecule 1. A mixture of 1c (50 mg) and 1d[1] (50 mg) in imidazole (5 g) was stirred for 3 hours at 140 °C under N_2. After cooling to room temperature, ethanol (50 mL) and concentrated HCl (12 N, 50 mL) were added to the mixture and stirred overnight. The resulting red solid was collected by vacuum filtration through a 0.45 μm membrane filter and rinsed thoroughly with water and ethanol. The residue was purified by column chromatography (silica, chloroform: acetone =100:1) to afford molecule **1 (**34 mg). ^{1}HNMR (300 MHz, $CDCl_3$) δ 8.65-8.62 (m, 4H), δ 8.55-8.52 (m, 4H), 7.24-7.15 (m, 4H), 6.97-6.87 (m, 2H), 6.87-6.78 (m, 1H), 6.76-6.72 (m, 1H), 5.37 (s, 2H), 5.00 (s, 2H), 4.19(t, J = 8.0 Hz, 2H), 3.76 (s, 3H), 1.77-1.54 (m, 2H), 1.44-1.25 (m, 18H), 0.87-0.84(m, 3H).

MALDI-TOF-MS: (m/z) =784.6.

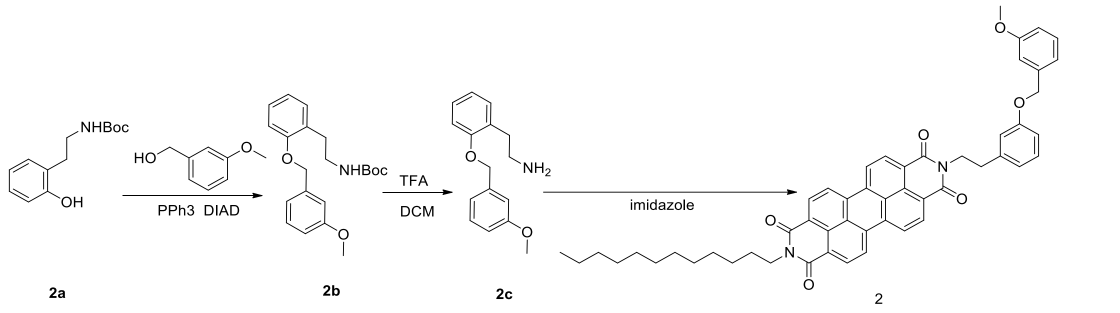

tert-butyl 2-((3-methoxybenzyl)oxy)phenethylcarbamate (2b). 2a (530 mg, 2.24 mmol), 3-Methoxybenzyl alcohol (309 mg, 2.24 mmol), DIAD (542 mg, 2.69 mmol), and PPh_3 (704 mg, 2.69 mmol) were added into 10 mL dichloromethane and stirred overnight at room temperature under N_2. Then the reaction was added with H_2O (10 mL) and the mixture was extracted with dichloromethane (3 × 10 mL). The combined organic layers were dried over sodium sulfate, and concentrated under vacuum. The residue was purified by column chromatography (silica, hexanes: ethyl acetate = 10:1) to afford 2b (420 mg).

^{1}HNMR (300 MHz, $CDCl_3$) δ 7.32-7.19 (m, 2H), 7.02-6.99 (m, 2H), 6.88-6.78 (m, 4H), 5.02 (s, 2H), 4.54 (br, 1H), 3.82 (s, 3H), 3.41-3.34 (m, 2H), 2.77 (t, J = 7.2 Hz, 2H), 1.44 (s, 9H).

2-(2-((3-methoxybenzyl)oxy)phenyl)ethanamine (2c). 2b (420 mg) was added to a solution of dichloromethane (5 mL) and trifluoroacetic acid (5 mL). The resulting solution was stirred for 3 h at room temperature, and then trifluoroacetic acid was removed by rotary evaporation. Saturated $NaHCO_3$ solution (10 mL) was added to the residue and the resulting solution was extracted with dichloromethane (3 ×10 mL). The combined organic layers were dried over sodium sulfate, and concentrated under vacuum to afford compound 2c**,** which was not purified and used directly for the following reaction.

Molecule 2. A mixture of 2c (50 mg) and 1d (50 mg) in imidazole (5 g) was stirred for 3 hours at 140 ^{o}C under N_2. After cooling to room temperature, ethanol (50 mL) and concentrated HCl (12 N, 50 mL) were added to the mixture and stirred overnight. The resulting red solid was collected by vacuum filtration through a 0.45 μm membrane filter and rinsed thoroughly with water and ethanol. The residue was purified by column chromatography (silica, chloroform: acetone =100:1) to afford molecule **2 (**30 mg). ^{1}HNMR (400 MHz, $CDCl_3$) δ 8.70-8.68 (m, 4H), δ 8.63-8.61 (m, 4H), 7.23-7.15 (m, 2H), 6.97-6.99 (m, 4H), 6.86-6.82 (m, 2H), 5.00 (s, 2H), 4.31 (t, *J* = 8.0 Hz, 2H), 4.19 (t, *J* = 8.0 Hz, 2H), 3.79 (s, 3H), 3.03 (t, *J* = 7.6 Hz, 2H), 1.77-1.72 (m, 2H), 1.44-1.29 (m, 18H), 0.88-0.84 (m, 3H).

MALDI-TOF-MS: (m/z) =798.6.

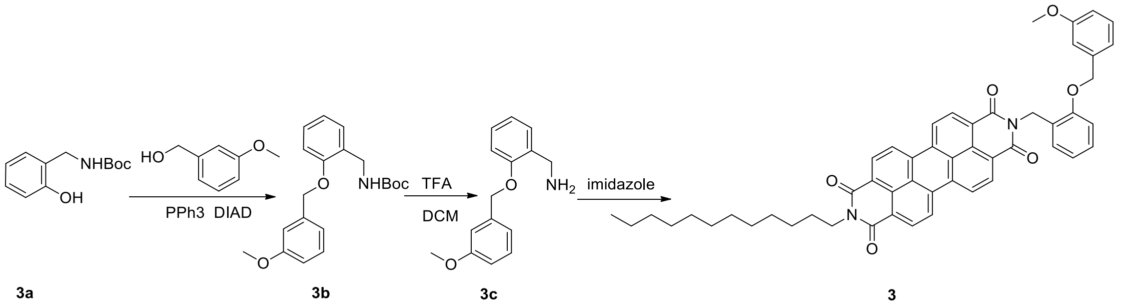

tert-butyl 2-((3-methoxybenzyl)oxy)benzylcarbamate (3b). 3a (500 mg, 2.24 mmol), 3-Methoxybenzyl alcohol (309 mg, 2.24 mmol), DIAD (542 mg, 2.69 mmol), and PPh_3 (704 mg, 2.69 mmol) were added into 10 mL dichloromethane and stirred overnight at

room temperature under N_2. Then the reaction was added with H_2O (10 mL) and the resulting solution was extracted with dichloromethane (3 × 10 mL). The combined organic layers were dried over sodium sulfate, and concentrated under vacuum. The residue was purified by column chromatography (silica, hexanes: ethyl acetate = 10:1) to afford 3b (520 mg).

^{1}HNMR (400 MHz, $CDCl_3$) δ 7.42-7.01 (m, 3H), 6.98-6.85 (m, 5H), 4.84 (br, 1H), 5.08 (s, 2H), 4.36 (s, 2H), 3.82 (s, 3H), 1.43 (s, 9H).

(2-((3-methoxybenzyl)oxy)phenyl)methanamine (3c) 3b (520 mg) was added to a solution of dichloromethane (5 mL) and trifluoroacetic acid (5 mL). The resulting solution was stirred for 3 h at room temperature, and then trifluoroacetic acid was removed by rotary evaporation. Saturated $NaHCO_3$ solution (10 mL) was added to the residue and the resulting solution was extracted with dichloromethane (3 ×10 mL). The combined organic layers were dried over sodium sulfate, and concentrated under vacuum to afford compound 3c**,** which was not purified and used directly for the following reaction.

Molecule 3. A mixture of 3c (50 mg) and 1d (50 mg) in imidazole (5 g) was stirred for 3 hours at 140 ^oC under N_2. After cooling to room temperature, ethanol (50 mL) and concentrated HCl (12 N, 50 mL) were added to the mixture and stirred overnight. The resulting red solid was collected by vacuum filtration through a 0.45 μm membrane filter and rinsed thoroughly with water and ethanol. The residue was purified by column chromatography (silica, chloroform: acetone =100:1) to afford molecule **3 (**32 mg). ^{1}HNMR (300 MHz, $CDCl_3$) δ 8.71-8.69 (m, 8H), 7.23-7.20 (m, 5H), 7.03-6.88 (m, 3H), 5.36 (s, 2H), 5.12 (s, 2H), 4.28 (t, J = 8.0 Hz, 2H), 3.77 (s, 3H), 1.77-1.54 (m, 2H), 1.44-1.25 (m, 18H), 0.89-0.85 (m, 3H).

MALDI-TOF-MS: (m/z) =784.6.

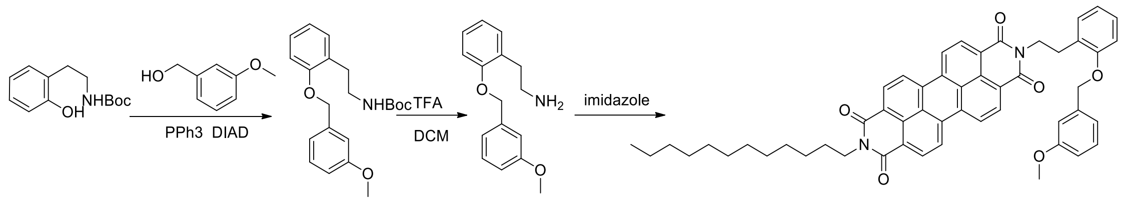

tert-butyl 2-hydroxyphenethylcarbamate (4b). 4a (530 mg, 2.24 mmol), 3-Methoxybenzyl alcohol (309 mg, 2.24 mmol), DIAD (542 mg, 2.69 mmol), and PPh_3 (704 mg, 2.69 mmol) were added into 10 mL dichloromethane and stirred overnight at room temperature under N_2. Then the reaction was added with H_2O (10 mL) and and the mixture was extracted with dichloromethane (3 × 10 mL). The combined organic layers were dried over sodium sulfate, and concentrated under vacuum. The residue was purified by column chromatography (silica, hexanes: ethyl acetate = 10:1) to afford 4b (360 mg).

^{1}HNMR (400 MHz, $CDCl_3$) δ 7.15-6.93 (m, 3H), 7.01-6.84 (m, 5H), 5.04 (s, 2H), 4.68 (br, 1H), 3.80 (s, 3H), 3.39-3.35 (m, 2H), 2.86 (s, t, J = 6 Hz, 2H), 1.41 (s, 9H)

2-(2-((3-methoxybenzyl)oxy)phenyl)ethanamine (4c) 4b (360 mg) was added to a solution of dichloromethane (5 mL) and trifluoroacetic acid (5 mL). The resulting solution was stirred for 3 h at room temperature, and then trifluoroacetic acid was removed by rotary evaporation. Saturated $NaHCO_3$ solution (10 mL) was added to the residue and the resulting solution was extracted with dichloromethane (3 ×10 mL). The combined organic layers were dried over sodium sulfate, and concentrated under vacuum to afford compound 4c**,** which was not purified and used directly for the following reaction.

Molecule 4. A mixture of 4c (50 mg) and 1d (50 mg) in imidazole (5 g) was stirred for 3 hours at 140 ^oC under N_2. After cooling to room temperature, ethanol (50 mL) and concentrated HCl (12 N, 50 mL) were added to the mixture and stirred overnight. The resulting red solid was collected by vacuum filtration through a 0.45 μm membrane filter and rinsed thoroughly with water and ethanol. The residue was purified by column

chromatography (silica, chloroform: acetone =100:1) to afford molecule **4 (**34 mg). ^{1}HNMR (400 MHz, $CDCl_3$) δ 8.65-8.63 (m, 2H), 8.53-8.47 (m, 6H), 7.24-7.23 (m, 3H), 6.94-6.74 (m, 5H), 4.96 (s, 2H), 4.50 (t, *J* = 7.2 Hz, 2H), 4.19 (t, *J* = 7.6 Hz, 2H), 3.76 (s, 3H), 3.18 (t, *J* = 7.2 Hz, 2H), 1.75-1.73 (m, 2H), 1.44-1.29 (m, 18H), 0.88-0.83 (m, 3H).MALDI-TOF-MS: (m/z) =798.6.

[1] Detection of Amines with Fluorescent Nanotubes: Applications in the Assessment of Meat Spoilage. Yanyong Hu, Xiaojie Ma, Yibin Zhang, Yanke Che, Jincai Zhao. ACS Sens. **2016**, *1*, 22-25.

Other supporting figures

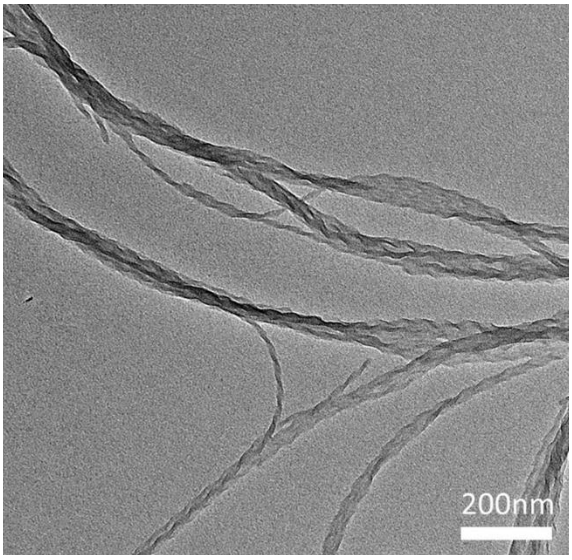

Figure S1. TEM image of nanocoils assembled from **1** at 6 h after beginning the self-assembly.

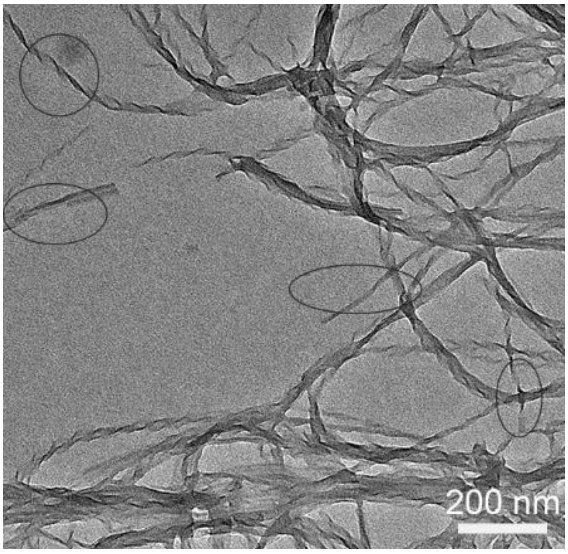

Figure S2. TEM image of the intermediate morphology with partly coiled structure assembled from **1** at 1 h after beginning the self-assembly process (indicated by circles).

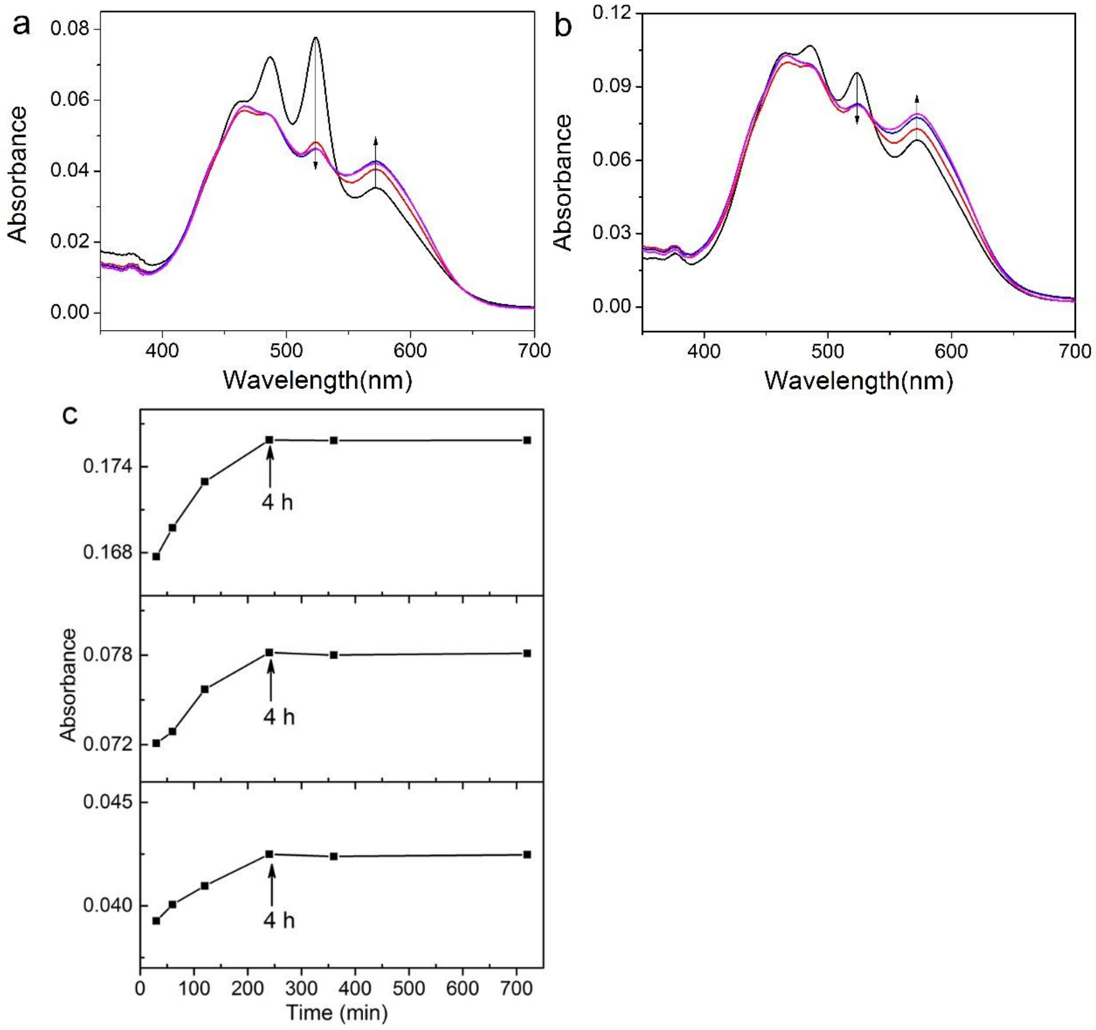

Figure S3. (a) Absorption spectra profile of **1** aggregates at different assembly time points after the injection of 0.3 mL of chloroform solution of **1** with0.13 mM into 4.5 mL of ethanol: 2 min (black), 1 h (red), 4 h (blue) and 6 h (magenta). (b) Absorption spectra profile of **1** aggregates at different assembly time points after the injection of 0.3 mL of chloroform solution of **1** with 0.26 mM into 4.5 mL of ethanol: 2 min (black), 30 min (red), 4 h (blue) and 6 h (magenta). (c) The time-dependent absorption changes at 573 nm of **1** aggregates formed upon injection of 0.3mL of chloroform solution of **1** with 0.65 mM (top); 0.26 mM (middle); 0.13 mM (bottom) into 4.5 mL of ethanol.

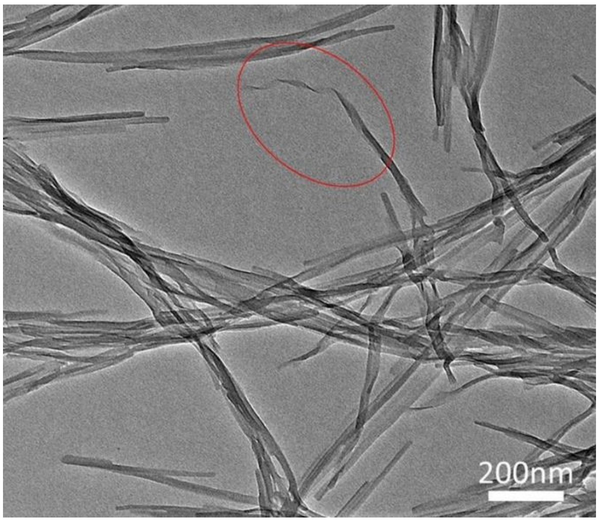

Figure S4. TEM image of the intermediate morphology with partly ribbon structure assembled from **2** at 1 h after beginning the self-assembly process (indicated by red circle).

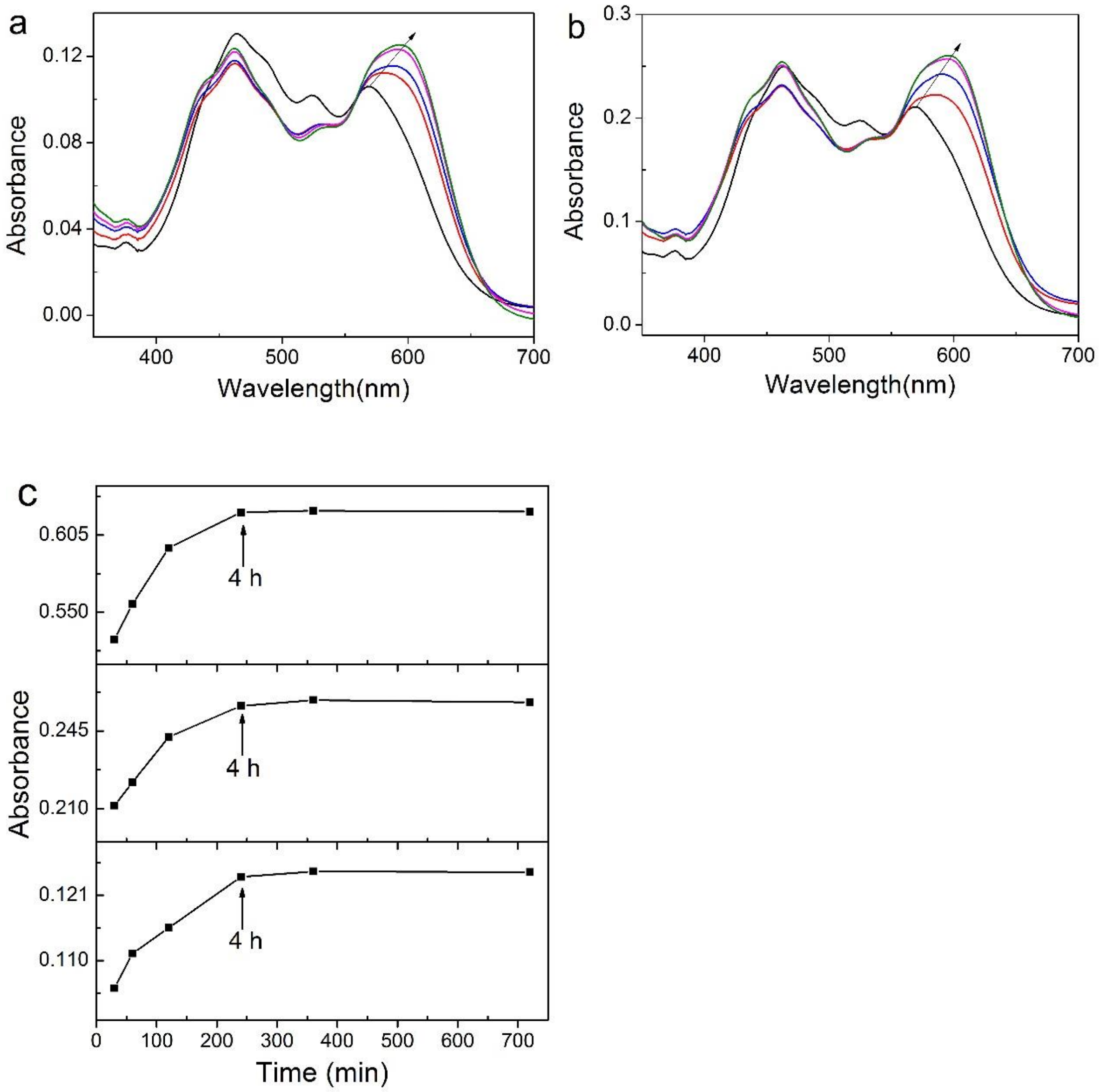

Figure S5. (a) Absorption spectra profile of **2** aggregates at different assembly times after the injection of 0.3 mL of chloroform solution of 2 with 0.13 mM into 4.5 mL of ethanol: 2 min (black), 1 h (red), 2 h (blue), 4 h (magenta) and 6 h (olive). (b) Absorption spectra profile of **2** aggregates at different assembly time points after the injection of 0.3 mL of chloroform solution of **2** with 0.26 mM into 4.5 mL of ethanol: 2 min (black), 1 h (red), 2 h (blue), 4 h (magenta) and 6 h (olive). (c) The time-dependent absorption changes at 590 nm of **2** aggregates after the injection of 0.3 mL of chloroform solution of **2** with 0.65 mM (top); 0.26 mM (middle); 0.13 mM (bottom) into 4.5 mL of ethanol.

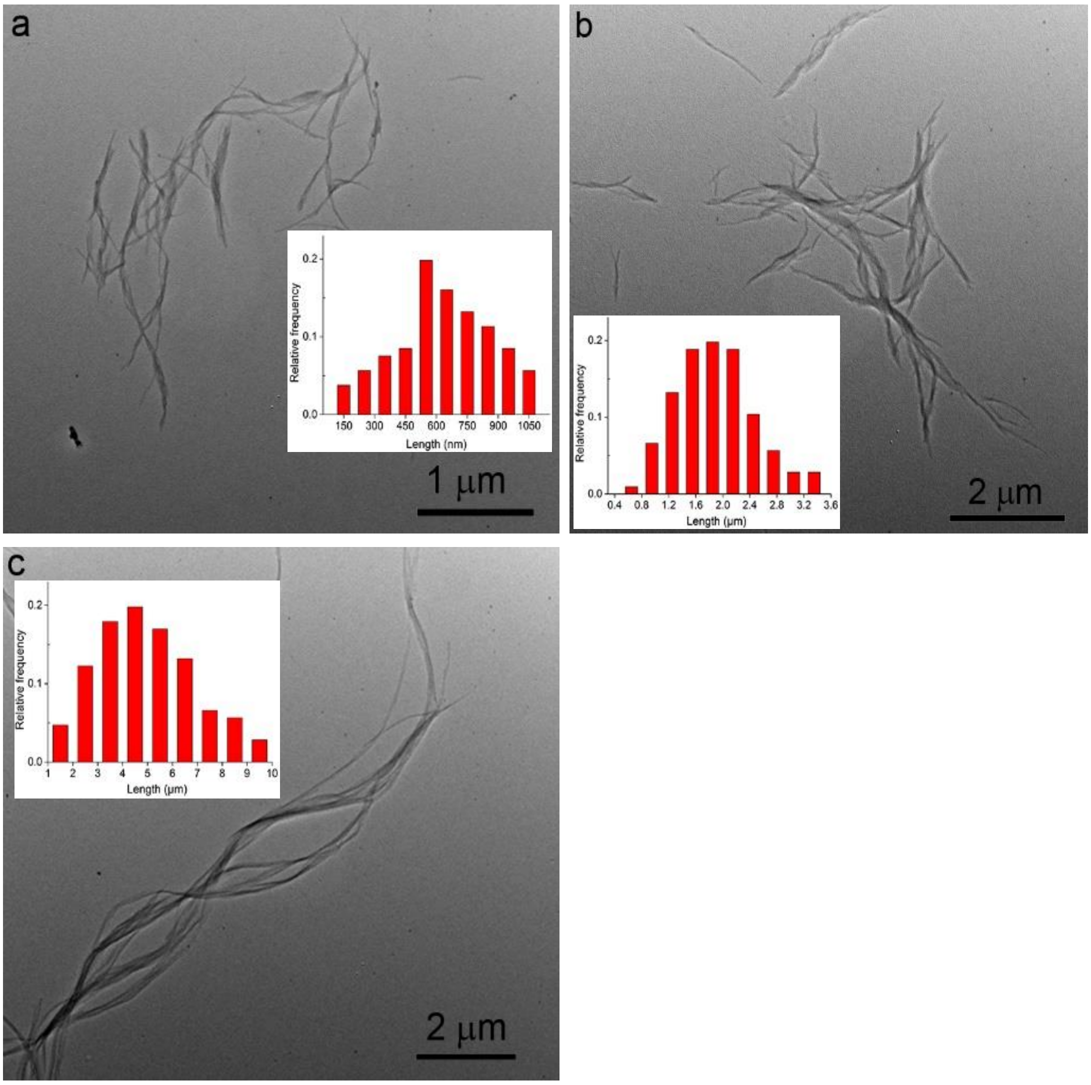

Figure S6. TEM imaging of the assemblies from **4** at different self-assembling time points: 5 min (a); 5 h (b); 24 h (c). Inset: length distribution of the formed assemblies (the length of more than 50 aggregates was measured), which clearly shows the aggregates elongated with time.

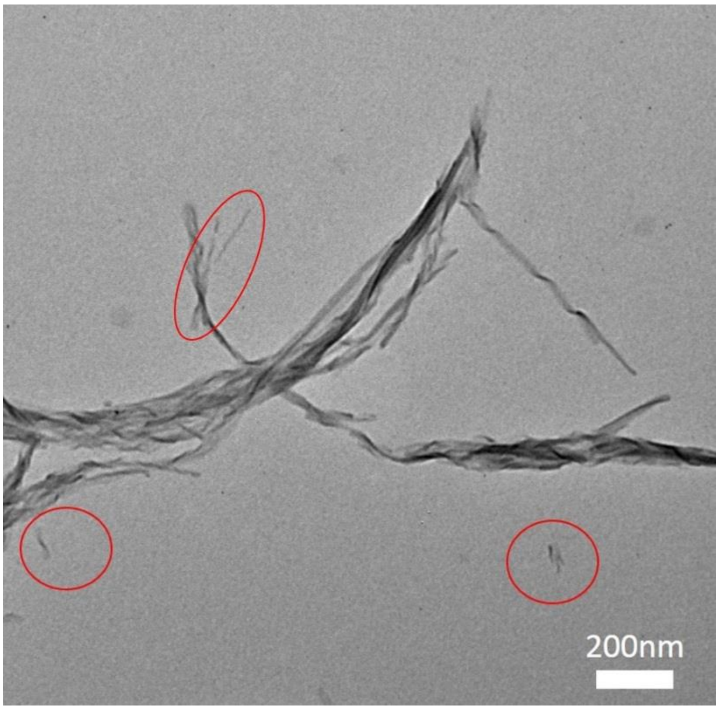

Figure S7. TEM image of the intermediate morphology with shorter nanocoils structure assembled from **4** at 1 h after beginning the self-assembly process (indicated by red circle).

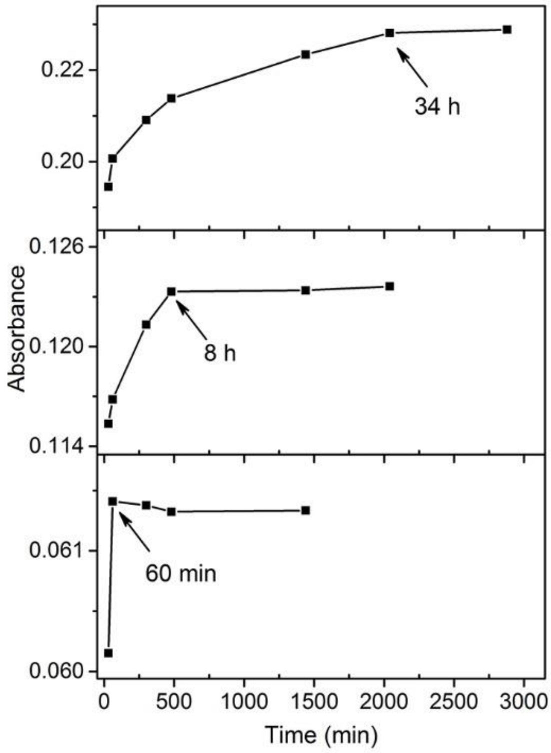

Figure S8. The time-dependent absorption changes at 570 nm of **4** aggregates formed upon injection of 0.3 mL of chloroform solution of **4** with 0.26 mM (top); 0.13 mM (middle); 0.06 mM (bottom) into 4.5 mL of ethanol.
